# Efficacy of Therapeutic Exercise in Reversing Decreased Strength, Impaired Respiratory Function, Decreased Physical Fitness, and Decreased Quality of Life Caused by the Post-COVID-19 Syndrome

**DOI:** 10.3390/v14122797

**Published:** 2022-12-15

**Authors:** Diego Fernández-Lázaro, Gema Santamaría, Nerea Sánchez-Serrano, Eva Lantarón Caeiro, Jesús Seco-Calvo

**Affiliations:** 1 Department of Cell Biology, Genetics, Histology and Pharmacology, Faculty of Health Sciences, Campus de Soria, University of Valladolid, 42003 Soria, Spain; 2 Neurobiology Research Group, Faculty of Medicine, University of Valladolid, 47002 Valladolid, Spain; 3 Department of Anatomy and Radiology, Faculty of Health Sciences, Campus de Soria, University of Valladolid, 42003 Soria, Spain; 4 Microbiology Unit of Soria University Assistance Complex (CAUSO), Santa Bárbara Hospital, Castille and Leon Health (SACyL), 42003 Soria, Spain; 5 Physiotherapy Group FS1, General Surgery Research Group, Galicia Sur Health Research Institute (IIS Galicia Sur), SERGAS-UVIGO, Faculty of Physical Therapy, University of Vigo, 36005 Ponteveda, Spain; 6 Department of Physiotherapy, Institute of Biomedicine (IBIOMED), Campus de Vegazana, University of León, 24071 León, Spain; 7 Department of Physiology, Faculty of Medicine, University of the Basque Country, 48900 Leioa, Spain

**Keywords:** SARS-CoV-2, post-COVID-19, long COVID-19, exercise, respiratory function, physical fitness, quality of life, fatigue

## Abstract

In the current global scenario, many COVID-19 survivors present a severe deterioration in physical strength, respiratory function, and quality of life due to persistent symptoms and post-acute consequences of SARS-CoV-2 infection. These alterations are known as post-COVID-19 syndrome for which there is no specific and effective treatment for their management. Currently, therapeutic exercise strategies (ThEx) are effective in many diseases by reducing the appearance of complications and side effects linked to treatment, and are consequently of great relevance. In this study, we review the effect of ThEX in reversing decreased strength, impaired respiratory function, decreased physical fitness, and decreased quality of life (QoL) caused by post-COVID-19 syndrome. A literature search was conducted through the electronic databases, Medline (PubMed), SciELO and Cochrane Library Plus for this structured narrative review for studies published from database retrieval up till 12 December 2022. A total of 433 patients with post-COVID-19 syndrome condition (60% women) were included in the nine studies which met the inclusion/exclusion criteria. Overall, post-COVID-19 syndrome patients who followed a ThEx intervention showed improvements in strength, respiratory function, physical fitness and QoL, with no exercise-derived side effects. Thus, ThEx based on strength, aerobic and respiratory training could be an adjuvant non-pharmacological tool in the modulation of post-COVID-19 syndrome.

## 1. Introduction

Coronavirus disease 2019 (COVID-19) represents one of the largest pandemics the world has ever faced and continues to produce global health, social and economic crises [1]. As of October 2022, more than 600 million have been infected and 6.5 million have died [2]). COVID-19 can be asymptomatic or cause a wide variety of clinical symptoms (Table 1). Eighty percent of patients present with mild or moderate symptomatology, but the remaining 20% develop moderately severe/severe symptoms associated with respiratory failure and acute respiratory distress syndrome (ARDS), related to Severe Acute Respiratory Syndrome Coronavirus 2 (SARS-CoV-2). Less frequently, COVID-19 triggers coagulation disturbances, septic shock, multi-organ failure, and complications secondary to systemic inflammation, which can lead to multi-organ failure and death [3,4,5,6]. 

**Figure 1:**
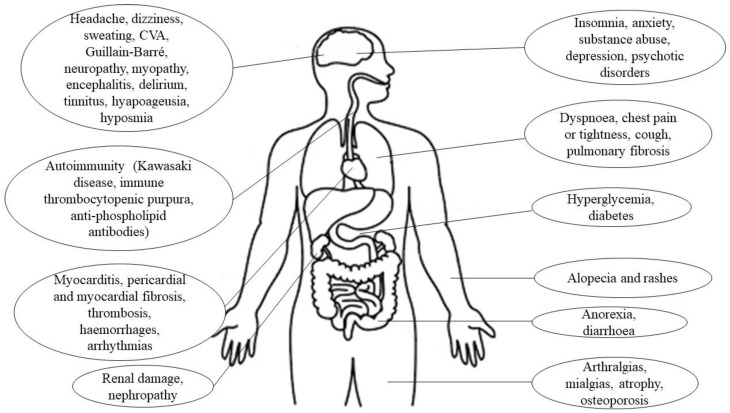
Sequelae caused by SARS-CoV-2 infection.

Some patients have reported post-acute manifestations of SARS-CoV-2 infection that appear as residual symptoms, due to the structural damage caused by the acute COVID-19 phase [3]. These post-COVID-19 sequelae are more common in elderly women who suffered severe complications during the acute infection process [14]. More than 200 sequelae have been reported, the most frequent being respiratory distress, alterations in taste and smell, fatigue, and neuropsychological symptoms such as memory loss, anxiety, or depression (Figure 1) [14,15,16]. Notably, only 65% of patients recover their pre-SARS-CoV-2 infection state of health within 2–3 weeks [17]. Although some post-COVID-19 sequelae usually resolve spontaneously, they can be very disabling, due to bed rest and the consequent reduction in daily activity, which significantly reduces physical capacity, increasing dyspnea and fatigue [4,18]. Additionally, as a consequence of increased secretions, the development of fibrosis and respiratory muscle weakness trigger alterations in the ventilatory pattern [19]. These alterations cause patients difficulties in persuing their work activities, fulfilling their family obligations, and even maintaining correct personal hygiene, which significantly diminishes their quality of life (QoL) [3,18].

It has recently been shown that a variety of symptoms can remain after acute SARS-CoV-2 infection and this condition is known as long COVID-19 (LC) [3]. The clinical presentation of the patient with LC is not compatible with post-COVID-19 because, although there is no fixed pattern in all patients, the symptomatology usually presents with flare-ups in which symptoms may worsen and new clinical manifestations appear [3]. The symptoms of LC may persist from the fourth week post-infection, lasting up to the twelfth week. There is a great variety of symptoms, the most frequent being fatigue, post-exertional malaise, dyspnea, headache and cognitive dysfunction that last for months after acute COVID-19 [3,14], cognitive and mental deficiencies, dry cough, chest and joint pain, palpitations, cardiac problems, myalgia, smell and taste dysfunctions, headaches and gastrointestinal problems [14]. Specifically, it has been reported that symptoms persist in 20% of those infected with SARS-CoV-2 at week 5 and in 10% at week 12, especially in women aged 36–50 years with previous comorbidities [3]. Associated risk factors may include gender (female sex), more than five early symptoms in the acute phase of COVID-19, early dyspnea, previous psychiatric disorders, and dysregulated specific biomarkers such as D-dimer, C-reactive protein (CRP), and lymphocyte count [3,14]. 

### 1.1. Therapeutic Exercise

**Figure 2:**
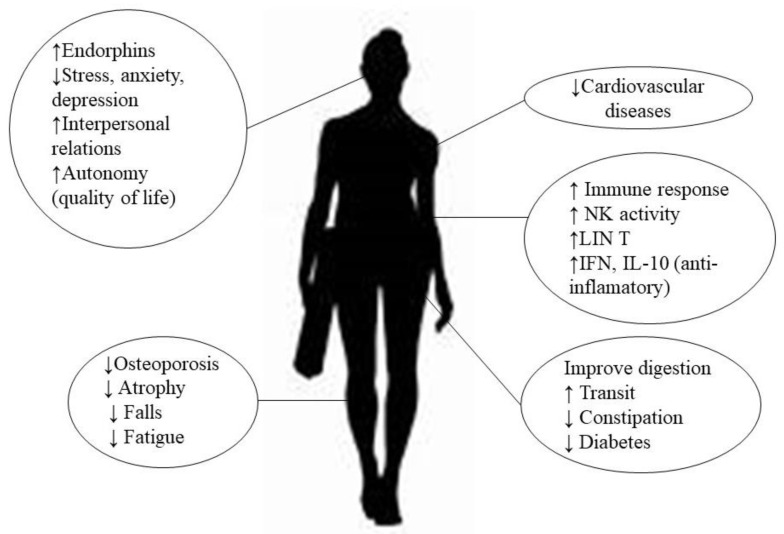
Potential benefits of physical activity.

Several studies [20,21,22] have described the effectiveness of therapeutic exercise (ThEx) as a non-pharmacological adjuvant therapy in breast cancer [22], chronic kidney disease [20], and inflammatory bowel disease [21]. For these patients, it has been observed that it not only improves the physical performance of patients, but also reduces the clinical signs of the diseases and induces psychological changes associated with both the disease and the treatment, leading to a better health-related QoL (HRQoL) [20,21,22]. In addition, other benefits associated with the practice of exercise have been described (Figure 2), such as a reduction in the risk of cardiovascular disease and better regulation of blood glucose levels, reducing the risk of developing diabetes [23,24,25,26,27]. At the level of the locomotor system, it prevents osteoporosis, muscle atrophy and falls, especially in the elderly, and reduces fatigue on exertion, increasing the capacity for oxygen utilization [23,24].

Exercise has an impact on the immune system, so that its moderate practice boosts the body’s immune response, reducing the incidence and severity of infectious processes, especially respiratory ones [28]. In this context, the increase in the cytotoxic activity of the Natural Killer (NK) and the stimulation of the proliferative capacity of T lymphocytes [28,29,30] could be among the mechanisms responsible for the observed benefits. In addition, regular exercise increases the plasma concentration of interferons (IFN-α, IFN-β, IFN-γ): these mediators mainly have an antiviral function by altering replication, stopping the process of gene expression and destroying the viral structure [28,31]; in addition, they increase the plasma concentration of interleukin-10 (IL-10) which—together with IFN-γ—act as anti-inflammatory cytokines [25,32,33], and may ameliorate the exacerbated inflammatory state caused by SARS-CoV-2. In general, exercise exerts immunomodulatory effects, controls the viral entry gate, modulates inflammation, stimulates nitric oxide synthesis pathways, and establishes control over oxidative stress mechanisms that allow control and modulation of SARS-CoV-2 infection [31]. However, intense and strenuous exercise can cause the opposite effect, reducing immune protection and increasing inflammation, so it is important to correctly control the intensity of exercise in its application as a therapeutic tool [25,28,30]. Nevertheless, exercise provides notable psychological and social benefits, derived from the release of endorphins by the hypothalamus and pituitary gland, in turn reducing stress, anxiety and depression [23,25,34,35]. On the other hand, it promotes interaction with other individuals and increases autonomy and QoL, especially in cases of disability such as post-COVID-19 and LC patients [23,35]. 

### 1.2. Post-COVID-19 Syndrome

Formally, the World Health Organization (WHO) considers the condition “post-COVID-19 syndrome” as the effects derived from clinical states of post-COVID-19 sequelae and/or LC. Post-COVID-19 syndrome could affect any person with a history of SARS-CoV-2 infection; and the signs or symptoms are maintained over time [36]. This disease is still poorly understood, and at present, there is no specific and effective treatment for the management of post-COVID-19 syndrome. Therefore, there is a need to seek to establish strategies to combat it, since it affects COVID-19 survivors at all levels of disease severity, including younger adults, children and people who are not hospitalized [37]. 

### 1.3. Exercise and Post-COVID-19

Exercise has been shown to be an effective therapy for most chronic diseases and microbial infections with preventive/therapeutic benefits, considering that exercise involves primary immune mediators and/or anti-inflammatory properties [14,38]. The implementation of ThEx programs in post-COVID-19 syndrome patients could be a useful complementary tool to stimulate recovery, improve QoL and provide immune protection in post-COVID-19 and LC patients. Specifically, ThEx programs that include strength routines, aerobic exercise, anaerobic exercise and specific respiratory medicine techniques such as ventilatory pattern reeducation and respiratory muscle training (RMT) could be the key to mitigate post-COVID-19 and LC [39]. Therefore, through the present narrative literature review, we set out to critically review the currently available evidence on the efficacy of different ThEx protocols in reversing the decreased strength, impaired respiratory function, decreased physical fitness and decreased QoL caused by post-COVID-19 syndrome.

## 2. Method

### 2.1. Search Strategy

The present study is a structured narrative review, conducted between May and December 2022, which aims to evaluate the impact of different exercise routines to modulate clinical/symptomatological alterations in post-COVID-19 and LC patients. The literature search was conducted through the electronic databases, Medline (PubMed), SciELO and Cochrane Library Plus. The search strategy included terms related to exercise, post-COVID-19, LC and the outcomes as well as a combination of these with Medical Subject Headings (MeSH) index: *Coronavirus, COVID-19, Long COVID-19, post-COVID-19, functional recovery, functional rehabilitation, exercise, aerobic exercise, anaerobic exercise, strength exercise, pulmonary physiotherapy, respiratory muscle training, inspiratory muscle training, QoL and dyspnea*, linked by the Boolean operators “AND” and “OR”. Titles (to identify duplicates) and abstracts were independently reviewed by two reviewers. The full text of relevant articles was obtained from database retrieval up till October 2022. Inclusion criteria were assessed independently for these two reviewers, and a third reviewer resolved any disagreement between them. Four additional records were obtained from reference lists in relevant articles.

### 2.2. Selection Criteria

We based selection of records on the following criteria: (a) adults with post-COVID-19 syndrome stemming from previous infection with SARS-CoV-2 (excluding animal and/or in vitro studies); (b) ThEx intervention studies to modulate the symptomatology and decrease the clinical outcome of post-COVID-19 modular syndrome; (c) systematic or narrative reviews, clinical trials, observational studies, or case studies (excluding editorials or letters to the editor); (d) studies that assessed as outcomes (primary, secondary or safety) any of the standard strength tests (handgrip test, Medical Research Council [MRC] muscle strength scale, 30 s sit-to-stand test [30-STS], Lower strength, Daniels strength test); respiratory function (forced expiratory volume in the first second [FEV1], forced vital capacity [FVC], FEV1/FVC, pulmonary diffusion of carbon dioxide [DLCO], MRC dyspnea scale, Maximal inspiratory pressure [MIP], sustained maximal inspiratory pressure [SMIP], Maximal expiratory pressure [MEP], inspiratory time, respiratory fatigue index [FITr], Barthel based on dyspnea [BID]; Transition Dyspnea Index [TDI]); physical fitness (6-min walk test [6MWT], Balke test, Time & Go, Short Physical Performance Battery [SPPB], Functional Ambulatory Category [FAC], walking speed); QoL (SF-36, EuroQoL, Sarcopenia QoL [SarQoL], Interstitial Lung Disease Quality of Life [K-BILD], EuroQol 5 dimensions 5 level [Eq-5D-5L]) biomarkers; and (e) studies with clear information on the intervention (type, methodology and duration) of ThEx. All records that did not meet these criteria were excluded.

### 2.3. Data Extraction

Two reviewers (D.F.-L. and J.S.-C.) scrutinized and synthesized data from all selected studies into a comprehensive table using a standardized data extraction. A third reviewer (G.S.G.) resolved all disagreements between them. Information extracted from the selected studies included the name of the first author; publication year; country where the study was conducted; study design; sample size; participants’ sex and age; ThEx intervention; type of training; duration of intervention; outcomes; and results.

## 3. Results

Table 2 and Table 3 summarize the information from the studies included in this review, indicating the author/s, year of publication and country, the characteristics of the sample investigated (Table 2), the exercise intervention, the variables analyzed and, finally, the results (Table 3).

### 3.1. Characteristics of the Participants and Interventions

A total of 433 patients with post-COVID-19 syndrome condition (60% women) were included in the nine studies [40,41,42,43,44,45,46,47,48] selected for this structured narrative review, with ages ranging from 37 years [42] to 69 [40] years. Respiratory muscle training (RMT) [40,42,43,45,46], skeletal muscle strength training [41,42,44,48] and aerobic training (AE) [41,42,44,48] were the exercise interventions performed in the included studies. Seven studies [41,42,44,45,46,47,48] employed concurrent training as the intervention tool: RMT, strength training and AE [42,45,46] or AE and strength training [41,42,44,47,48]. The initiation of ThEx interventions occurred after overcoming the acute phase of SARS-CoV-2 infection [40,41,42,43,44,46,47,48], mild [42,43,44], moderate [40,47,48], severe/serious [41,46,48], in a period after SARS-CoV-2 infection of between 20 days [45] and 6 months [40]. Only Pancera et al. [45] started ThEx treatment in their only patient in the active phase of SARS-CoV-2 infection who required hospitalization in the intensive care unit (ICU). Regarding duration, ThEx periods ranging from 10 days [48] to 70 days [41] were implemented, with the number of weekly training sessions ranging from 2 [40,42,46] to 7 [48] (Table 2).

### 3.2. Outcome Evaluation

#### 3.2.1. Strength

Strength was evaluated in seven studies [41,42,44,45,46,47,48]. Substantial improvements in strength were obtained between the start and end of the ThEx program in all three clinical cases evaluated [41,42,47]. Furthermore, in the randomized controlled clinical trial conducted by Nambi et al. [44], a trend of improvement was observed in both low intensity (LI) and high intensity (HI) ThEx interventions. These authors [44] described a significant increase in strength (*p* < 0.05) in the LI group compared to the HI group. For the three cohort studies [45,46,48] included in this narrative review, significant improvements (*p* < 0.05) were observed in manual muscle testing via hand-held dynamometry [46], and in lower extremity strength [46,48] or six muscles in the upper and lower limbs on both sides assessed by the Medical Research Council (MRC) muscle strength scale [45], from the baseline to the end of the ThEx intervention period (Table 3).

#### 3.2.2. Respiratory Function

Three [40,43,45] of the studies included in this narrative review analyzed respiratory function after ThEx. Significant improvements (*p* < 0.05) were observed in the intervention group (IG) of all respiratory parameters studied in respiratory function [40] (forced expiratory volume in the first second [FEV1], forced vital capacity [FVC], FEV1/FVC, pulmonary diffusion of carbon dioxide [DLCO]) and inspiratory muscle strength [43] (maximal inspiratory pressure [MIP], sustained maximal inspiratory pressure [SMIP], inspiratory time, and respiratory fatigue index [FITr]). Furthermore, these improvements were significant (*p* < 0.05) in FEV1, FVC, FEV1/FVC, MIP and SMIP in the IG with respect to the control group (CG). In addition, both respiratory pressures, MIP and maximal expiratory pressure (MEP) improved after 25 days of exercise intervention in a patient who suffered severe COVID-19 and ARDS [45]. With diaphragmatic muscle reduction work, Mayer et al. [42] allowed attenuation of dyspnea (MRC dyspnea scale) (Table 3).

#### 3.2.3. Physical Capacity

Table 2 described improvements in physical capacity in the five studies evaluating it [40,41,42,45,48]. Lui et al. [40] showed a significant increase (*p* < 0.05) in the IG from the beginning of the study, and a significant improvement with respect to the CG in the 6-min walk test (6MWT) after 6 weeks of ThEx. In the study conducted by Udina et al. [48], significant improvements (*p* < 0.05) were observed in the Short Physical Performance Battery (SPPB), ambulation ability (Functional Ambulatory Category [FAC]) and gait speed in hospitalized and ICU patients. Improvements were also described in studies involving a single patient on the 6MWT [42], Time-up and go test [41,42], EuroQol questionnaire [45], cardiopulmonary capacity (Balke test) [41], range of motion (ROM) [47], and SPPB [45] (Table 3).

#### 3.2.4. Quality of Life

Four studies show improvements in the QoL [40,42,43,44] of patients after ThEx, being significant (*p* < 0.05) in the study conducted by Liu et al. [40] assessed by the SF-36 questionnaire. Furthermore, in this study [40], the QoL was significantly improved (*p* < 0.05) compared to the condition without exercise. Structured LI and HI exercise programs show substantial increases in the Sarcopenia QoL (SarQoL) [44] after 8 weeks of AE and strength training. In the case of LI interventions, these obtained significantly (*p* < 0.05) better scores than those of HI in the SarQoL of post-COVID-19 sarcopenic patients [44]. In addition, significantly (*p* < 0.05) better ratings were obtained on the King’s interstitial lung disease (inflammation and fibrosis) QoL questionnaire (K-BILD) for the IG after 8 weeks of RTM compared to the CG [43]. In the case of a patient with mild COVID-19 without previous hospitalization, his QoL was improved on all five dimensions (mobility, self-care, usual activities, pain/discomfort and anxiety/depression) of the questionnaire (EQ-5D-5L) [42] (Table 3).

#### 3.2.5. Other Biomarkers

ThEx programs in post-COVID-19 syndrome patients decrease the impact of fatigue [41], obtain significant improvements (*p* < 0.05) in the psychological aspect (anxiety and depression) [40], achieve a significant decrease (*p* < 0.05) of kinesiophobia (fear of performing movements that cause pain or that may worsen a previous injury, limiting certain activities) [44], and the reduction of pain intensity evaluated by numeric pain scale [47]. Body mass index (BMI) and quadriceps circumference were improved after 25 days of ThEx in a patient who was hospitalized in ICU and required mechanical ventilation [45]. No adverse effects related to exercise, RMT, strength training and/or AE training were reported in the nine registries selected [40,41,42,43,44,45,46,47,48] for this study (Table 3).

## 4. Discussion

The SARS-CoV-2 coronavirus pandemic has led to a multiplication of initiatives from sports, social, and health fields with the aim of stimulating exercise promotion in the multiple scenarios in which the COVID-19 global health crisis has been experienced. In the face of the massive bombardment of information and proposals of different types, this narrative review aimed to critically evaluate the effects of non-pharmacological therapeutic intervention through structured exercise programs on strength, respiratory function, physical capacity and QoL in adults with post-COVID-19 syndrome. Nine studies met the pre-specified inclusion/exclusion criteria. Overall, post-COVID-19 syndrome patients who followed a ThEx intervention, based on RMT [40,42,43,45,46], muscle strength training [41,42,44,45,46,47,48] and/or EA [41,42,44,45,46,47,48] showed improvements on strength [41,42,44,45,46,47,48], respiratory function [40,43,45], physical fitness [40,41,42,45,48] and QoL [40,42,43,44], with no ThEx-derived side effects.

### 4.1. Strength

Loss of muscle mass and function (sarcopenia) is a common occurrence among older adults and in other patients, regardless of age, with diseases that are accompanied by muscle wasting (cancer, heart failure, chronic kidney disease, liver cirrhosis, chronic obstructive pulmonary disease), multimorbidity and infections [49]. SARS-CoV-2 infection causes muscle damage with skeletal muscle involution and decreased muscle strength, thereby increasing disability and decreasing QoL [50]. This musculoskeletal loss, associated with SARS-CoV-2 infection, could be of multifactorial etiopathogenesis derived from inactivity, generalized inflammation, myalgias and arthralgias, insufficient energy and macronutrient intake, and hospital complications [51,52,53].

Muscle wasting is associated with systemic inflammatory hyper-response related to SAR-Cov-2 infection, with an exacerbation in the production of pro-inflammatory interleukins (IL)—IL-6, IL-8 and tumor necrosis factor [TNF-α]—increasing catabolism and oxidative stress, and causing severe myocyte damage [52,53,54]. In addition, the inflammatory status induces reduced rates of protein synthesis, in parallel with increased protein degradation, which explains the loss of muscle mass, and is activated by nuclear factor transcription (NF-kB). NF-kB is activated in response to viral and bacterial infection stimuli, increased IL-6, IL-8, TNF-α and oxidative stress [55].

Complications derived from hospitalization increase the magnitude of muscle loss range from 20% due to reduced immune response and risk of nosocomial infection up to 40% of pneumonia [55]. Forty percent of those infected by SARS-CoV-2 suffer myalgias (the thirrd most frequent symptom) and 15% arthralgias [52,53], which substantially reduce mobility and contribute to the degree of muscle atrophy. In fact, bed rest during hospital stay leads to a muscle wasting of 2% of muscle mass and 12.5% of muscle strength after 10 days of hospitalization [51]. Other COVID-19 symptoms are anorexia, nausea and vomiting, with insufficient energy and macronutrient intake, especially protein, to restore muscle tissue damage [52]. This situation of malnutrition could increase in those patients who required treatment in ICU with mechanical ventilatory support [56].

In subjects with COVID-19, some studies [12,53,57,58], reported a decrease in hand grip strength (HgS), a key system for the diagnosis of sarcopenia. In this context, Tuzun et al. [53] observed that HgS values during and after infection are lower than reference values, moreover, they were significantly lower in women than in men. Tanriverdi et al. [12] reported a reduction in HgS by 39.6% and in quadriceps strength in 35.4% of patients. Moreover, Johnsen et al. [57] have indicated that 28% of patients are below the 25th percentile of HgS. Meanwhile, a study with 73 patients with post-COVID-19 syndrome observed a decrease in quadriceps and biceps brachii strength below the expected 80% in 86% and 73% of patients, respectively [58]. For all these reasons, it is considered essential to prevent the loss of muscle mass and function at an early stage. However, due to the high rate of contagion, contact with COVID-19 positive patients was reduced to a minimum and exercise was considered appropriate only in patients who showed good tolerance to effort, delaying its use in patients with active infection until their symptoms had disappeared [59]. This therefore led us to perform late ThEx interventions.

**Figure 3:**
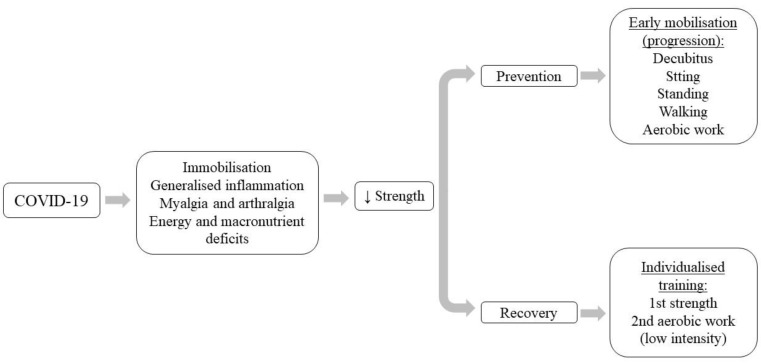
Implications of muscle strength in post-COVID-19 syndrome.

Our results showing increased strength after muscle strengthening exercises [41,42,44,45,46,47,48] corroborate the need to include muscle strength training in post-COVID-19 patients (Figure 3). It was considered that strengthening training would stimulate hypertrophic processes derived from the activation and proliferation of satellite cells [60], increased muscle actin mRNA expression, and increased protein synthesis, all modulated by the action of insulin-like growth factor type 1 (IGF-I), which increases in response to changes in the overload state of skeletal muscles [61]. It is important to know, given the peculiarities of the post-COVID-19 patient, that LI exercise have shown significant strength improvements compared to HI exercise [44]. This would indicate the need for ThEx interventions tailored to the patient’s clinical situation, physical condition, and tolerance to exercise [48]. Early-onset ThEx interventions during the active infection phase of SARS-CoV-2 could, in addition to strength gains, stimulate improvements in BMI and muscle mass gain [45], which could delay muscle atrophy in post-COVID-19 syndrome. Even in these early situations, it might be more beneficial to start with passive, active-assisted and/or active kinesitherapy exercises [62], strength training with therapist intervention and progressively including AE [63], which would potentially prevent muscle atrophy and capsule-ligamentous retractions.

Overall, this situation of wasting, and loss of muscle mass and function in post-COVID-19 syndrome makes it necessary to start early programs that include ThEx based on mobilization exercises in decubitus, sitting, sitting balance, transfers from sitting to standing, walking, upper and/or lower extremity cycloergometer training and strengthening exercises [64].

### 4.2. Respiratory Function

Alterations in respiratory function in post-COVID-19 syndrome are to a greater extent effects due to the sequelae of a severe acute disease rather than a LC process in the strict sense [59]. In fact, there is a decrease in respiratory parameters that is prolonged over time, and the reduction translates into the appearance of dyspnea, which is one of the most disabling symptoms of this disease, significantly reducing the QoL [43]. Thus, pulmonary performance is caused by restrictive and obstructive ventilatory patterns that are most frequent in patients who were admitted to the ICU with intubation and mechanical ventilation [19,43]. Restrictive problems may be due to the development of pulmonary fibrosis, due to increased expression of fibrosis-associated genes, and weakness of the respiratory musculature [19,64], as a consequence of SARS-CoV-2 infiltration in the myocytes of the diaphragm and/or mechanical ventilation [65]. Obstructive problems are caused by excess secretions, mucus and sputum, caused by SARS-CoV-2 pneumonia [62]. The reduction in respiratory parameters at hospital discharge due to the sequelae of the acute phase of COVID-19 was 47.2% in DLCO, and 25% had a decrease in total lung capacity of 65%, or 79% in cases of severe pneumonia. In addition, 13.6% of post-COVID-19 patients had markedly decreased values of FEV_1_ and 10% of FVC [66]. This decrease in respiratory parameters is prolonged over time, demonstrated by the fact that at 3 months post-infection, 68% of patients have values below 80% of the expected DLCO, and even lower in patients who required oxygen therapy during the acute phase of SARS-CoV-2 [57]. This reduction results in the appearance of dyspnea [67].

To improve pulmonary function in post-COVID-19 patients, different respiratory rehabilitation/recovery strategies have been developed with the aim of eliminating pulmonary secretions, reeducating the ventilatory pattern and increasing the strength of the respiratory musculature [62,63,68]. Pulmonary secretion clearance by pulmonary drainage allows mobilization of secretions from the middle and distal airways to the proximal airways, facilitating expectoration by increasing expiratory airflow velocity, thus preventing a decrease in ventilatory capacity and the generation of excessive and ineffective coughing [69]. Successful instruction of respiratory reeducation allows the post-COVID-19 syndrome patient to perform diaphragmatic breathing and to achieve a correct coordination between inspiratory and expiratory muscles [68]. RMT can achieve an increase in respiratory muscle strength [70]. In this regard, Curci et al. [71] followed the strategy of recovery of pulmonary function in post-COVID-19 patients with drainage techniques and reeducation of breathing with work on the coordination of the respiratory muscles of the thorax and abdomen for those who presented inspired oxygen fraction (FiO_2_) of 40–60%, and RMT that included exercises of thoracic expansion, forced inspiration/expiration, work with the incentive spirometer and positive pressure valves aimed at patients with a FiO_2_ between 20–40% [71].

**Figure 4:**
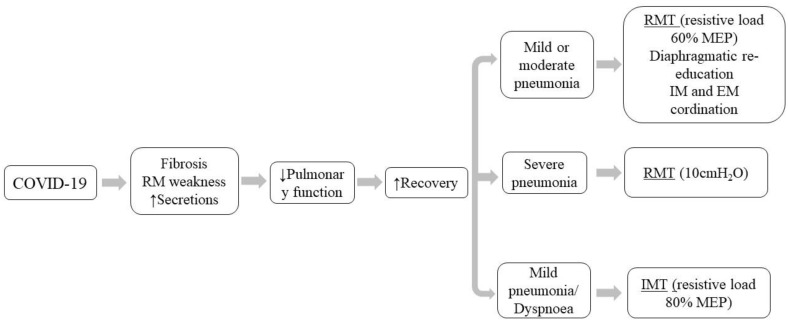
Respiratory muscle training in post-COVID-19 syndrome.

Three studies [40,43,45] included in this review show increases in respiratory function after RMT programs, which contributes to reaffirming the effectiveness of the different RMT in patients with post-COVID-19 syndrome (Figure 4). In clinical trials with RMT with a threshold device (whereby resistive load generates positive pressure during exhalation that helps to open the airways), compared to the CG, the IG showed significant improvements in FEV_1_, FVC, FEV_1_/FVC, DLCO [40] and inspiratory muscle training (IMT) with the PrO2TM (PrO2Fit Health Incorporated, RI, USA)—inspiratory flow resistive load—on MIP; moreover, SMIP [43] occurred at resistance loads of 60% of MEP [40] and 80% of MIP. These resistive workloads are well above physically active healthy adults who employ only 15% of MIP to obtain significant improvements in FEV_1_, FVC, FEV_1_/FVC, MEP (20–30%), and MIP (54%) [70]. Improvements in respiratory function through RMT are considered to be as a consequence of developing greater strength of the intercostal and/or abdominal respiratory muscles to generate an optimal contraction that thereby allow: sufficient ventilation and increase ventilatory efficiency, hypertrophy of the diaphragm, modification of muscle fiber composition towards type I, increase of type II fibers of intercostal musculature, optimization of the neuro-motor control of the respiratory musculature, and the generation of pressure to be maintained with a lower motor impulse and greater economy of the respiratory musculature [70]. RMT could be complemented with targeted breathing to help reeducate the ventilatory pattern, to obtain better results in respiratory function [40].

### 4.3. Fatigue and Physical Capacity

Fatigue is the debilitating and permanent subjective sensation of physical and/or mental tiredness characterized by lack of energy, muscle weakness, slow reactions, drowsiness and concentration deficit [72]. The etiology of fatigue in post-COVID-19 syndrome, although still under study, could be due to neuro-inflammation processes, vagus nerve involvement, mitochondrial dysfunction, oxidative stress, and viral persistence [3]. VanHerck et al. [73] indicated that 85.4% of patients report severe fatigue at three months post-COVID, being the most frequent sequel. Two types of fatigue have been established after post-COVID-19 infection: cognitive or mental fatigue, and neuromuscular or physical fatigue [72]. Mental fatigue affects vigilance, attention, working memory, judgment, and long-term memory, resulting in a perception of greater effort in activity. The inflammatory process reduces the amount of gamma amino butyric acid (GABA) receptors, causing an imbalance between dopaminergic and GABAergic transmitters, causing cognitive fatigue. In addition, alterations are produced in the frontal-subcortical circuit involved, reducing motivation [72]. Post-COVID-19 inflammation affects the central nervous system by altering sensitivity to pain and sleep [74]. Sleep disturbances affect 50% of patients with post-COVID-syndrome, contributing to fatigue [12]. Physical fatigue could be caused by a progressive failure of the peripheral nervous system (e.g., neuropathies, myopathies and Guillain–Barré syndrome) or central nervous system (e.g., reduced motor cortical excitability) [72].

The consequence of fatigue (physical and mental) are considered by patients as the main barrier to pre-infection activities due to reduced cardiopulmonary and musculoskeletal capacity [42,67,75]. In fact, the distance covered in the 6MWT remains reduced at 3- and 12-months post-infection in 70–80% of patients [4]. The British Thoracic Society has determined that 95% of patients with this symptom will need therapy to improve health-related fitness qualities such as cardio-respiratory endurance and muscular strength-endurance [76]. The different types of exercise intervention—RMT [40,42,43,45,46], strength training [41,42,44,45,46,47,48] and/or AE [41,42,44,45,46,47,48] (especially LI)—established improvements in physical fitness [40,41,42,45,48] in patients with post-COVID-19 syndrome based on the measured effects of ThEx on the cardiovascular system, namely improvement: of myocardial function and anti-arrhythmogenic effect; in the respiratory system: by strengthening the respiratory musculature and delaying the metaboreflex; and on the locomotor system by increasing muscle mass and strength resulting in greater physical fitness with better tolerance to effort [59,77]. These were demonstrated by the different tests evaluated SPPB [42,45], Time-up and go [41,42], Balke [41], FAC [48], and 6MWT [40,42]. These improvements in physical condition would contribute to a decrease in fatigue [21].

### 4.4. Quality of Life

One of the main characteristics of post-COVID-19 syndrome is the disability generated by its symptoms, which alter functionality to the point of incapacitating the performance of normal tasks. In fact, in Spain, approximately 70% of patients affected by post-COVID-19 syndrome were disabled in activities of daily living (ADL) such as cleaning the house, attending to family obligations and leisure [59]. This disability had an influence on QoL, where 50% experienced a significant decrease in QoL (EuroQol) [75], and 20% did not return to work after 12 months post-infection [4]. From all this, we can deduce the need to resume their QoL, and at the same time, reduce the demands they make on the health system to achieve this. To this end, it is essential to collect the patient’s experience and the opinion of those affected by means of scales or health measurement questionnaires to support comprehensive care for post-COVID-19 syndrome patients.

As a whole, ThEx based on RTM, strength training and AE allowed significant improvements in the QoL [40,42,43,44] of patients assessed by SF-36 [40], SarQoL battery [44], K-BILD-19 and EQ-5D-5L) [42]. In addition, exercise provided a significant improvement in the psychological aspect, a significant decrease in kinesiophobia and a substantial reduction in pain, evaluated by a numeric pain scale. This could imply adaptability and tolerance to exercise and possible recovery of ADLs that had been affected by the disease. The reported improvements in QoL derive from the physical optimization, physiological restoration and biological modulation achieved by exercise as an adjuvant therapy for post-COVID-19 syndrome. In addition, the psychological improvements have positive effects, derived from exercise, and could provide a coping strategy for those yearning to be a healthy person in the eyes of others, and help to overcome the health challenge. In addition, they help to reduce and eventually eliminate the potential for work, family, and social isolation experienced when given the impossibility of predicting how long the post-COVID-19 syndrome will last.

### 4.5. Telemedicine

Telemedicine refers to the use of telecommunications to provide specialist-supervised rehabilitation while the patient remains at home [78]. Telemedicine programs through mobile applications, virtual reality and wearable devices can play a vital role in post-discharge follow-up after COVID-19 [78], while minimize the risks associated with direct contact. Indeed, a prospective surveillance model (PSM), based on sensitive and user-friendly assessment tools, has been proposed for use by rehabilitation professionals in the management of patients requiring rehabilitation after COVID-19, which contemplates face-to-face as well as telematic follow-up of the patient [39]. These programs are especially indicated for people in whom difficulty with ADLs persists after completing face-to-face rehabilitation, with the aim of maintaining and increasing the autonomy achieved [79]. The recommended duration is about 12 weeks, with a frequency of 5–7 days per week [64]. The areas of intervention are mainly psychological support, nutritional advice and ThEx, including work on mobility, strength training, balance, coordination, AE, gait reeducation and RMT [43,64,79]. The main advantages of telemedicine are portability, versatility and low cost [64,80]. Additionally, the use of screens increases adherence to treatment thanks to the visual feedback received by the patient [64]. Finally, telerehabilitation makes it possible to provide adequate psychological support and to prescribe ThEx in the event that the patient must remain isolated [78].

Telemedicine programs have demonstrated benefits in the reduction of dyspnea, improvement of functional capacity and QoL similar to those obtained with traditional intervention [80]. A systematic review has reported improvements in 6MWT and dyspnea following breathing exercises or ThEx compared to the CG [81]. Additionally, a randomized clinical trial [43], included in this review, used exercise by IMT-based telemedicine for 8 weeks, with 3 weekly sessions of 20 min in which they performed 6 sets of 6 breaths while maintaining a resistance greater than 80% SMIP and achieved an increase in the dyspnea, activity and psychological subdomains of K-BILD, an increase in MIP and SMIP, and improved VO_2_max [43].

### 4.6. Considerations on Therapeutic Exercise

Although no adverse events resulting from exercise have been reported [40,41,42,43,44,45,46,47,48], we consider that ThEx should be used in patients who show good tolerance to exertion, and it may well be necessary to delay the use of exercise in patients who are severely affected or who present active infection with severe symptoms, until their health has improved. Furthermore, for ThEx to have beneficial effects, it must be performed under medical prescription and must comply with certain conditions regarding the type, frequency, duration, and intensity of exercise, which are aimed at improving health-related qualities of physical condition.

## 5. Conclusions

SARS-CoV-2 infection causes serious alterations in muscle strength, respiratory function and physical capacity causing fatigue resulting in reduced QoL. Although there is currently no specific and effective treatment for post-COVID management, ThEx (such as AE, muscle strength training, and RMT) has shown significant increases in muscle strength, lung function, physical fitness and decreases in fatigue, which substantially improve patients’ QoL. In addition, at this time of social isolation and distancing, telerehabilitation has shown similar benefits to face-to-face therapy, and is therefore a very good therapeutic option.

## Figures and Tables

**Table 1:** Clinical manifestations in patients infected with SARS-CoV-2.

Location	Clinical Manifestations
Respiratory System [6]	Cough, sore throat, dyspnea, pneumonia, bilateral interstitial inflammation, acute respiratory distress syndrome, rhinorrhea
Central nervous system [7]	Stroke, meningitis, encephalitis, headache, dizziness, ataxia, convulsions, confusion, hallucinations
Peripheral nervous system [7]	Hypoageusia, hiposmia/anosmia, neuralgia, Guillain–Barré syndrome, chemosensory dysfunction, hyporeflexia, stiffness
Endocrine system [8]	Hyperglycemia, ketoacidosis, adrenal insufficiency, thyrotoxicosis
Cardiovascular system [9,10]	Myocarditis, cardiac failure, acute myocardial infarction, cardiomyopathy, shock, arrhythmias, pulmonary thromboembolism, coagulation disorders, hypertension, palpitations
Digestive system [5,9]	Anorexia, nausea, vomiting, diarrhea, abdominal and epigastric pain, hepatic and pancreatic pathology
Excretory system [9,11]	Acute renal damage, tubular necrosis, nephropathy, proteinuria, hematuria
Locomotor system [12]	Rhabdomyolysis, mialgias, generalized weakness, fatigue, arthralgias, decreased bone density, osteonecrosis
Immune system [11]	Fever, lymphopenia, decreased CD4 and CD8, IL-10 and TNF- α, increased pro-inflamatory cytokines
Lymphatic system [9]	Mediastinal lymphadenopathy
Reproductive system [13]	Orchitis, scrotal discomfort, scrotal pain, infertility
Integumentary system [1]	Vesicular rash, maculopapular rash, urticarial rash, petechiae, acral lesions, livedoid lesions

**Table 2:** Design and participants of studies included in the review of the effect of physical activity on strength, respiratory function, fatigue and quality of life in post-COVID-19 and LC adults.

First Author, Year of Publication and Country	Design	Participants (Size and Characteristics of the Initial Sample)
Liu et al. [40], 2020, China	Controlled randomized clinical trial	n: 72 Moderate COVID-19 (hospitalization) *CG*: n: 36; 25 ♂, 11 ♀ Age (mean ± SD): 68.9 ± 7.6 y BMI (mean ± SD): 22.9 ± 3.9 kg/m^2^ *IG*: n: 36; 24 ♂, 12 ♀ Age (mean ± SD): 69.4 ± 8.0 y BMI (mean ± SD): 23.1 ± 3.5 kg/m^2^ Post COVID-19 ≥ 6 months
Longobardi et al. [41], 2022, Brasil	Case report	n: 1 ♀ Critical COVID-19 (71 days of hospitalization, with 49 days in ICU and invasive mechanical ventilation) Age: 67 y BMI: 27.1 kg/m^2^ Post COVID-19 ≥ 3 months
Mayer et al. [42], 2021, USA	Case report	n: 1 ♀ Mild COVID-19 (No hospitalization and no oxygen therapy) Age: 37 y Post COVID-19 ≥ 6 wk
McNarry et al. [43], 2022, United Kingdom	Controlled randomized clinical trial	n: 148 Mild COVID-19 (dyspnea) *CG*: n: 37; 2 ♂, 35 ♀ Age (mean ± SD): 46.13 ± 12.73 y BMI (mean ± SD): 27.81 ± 5.83 kg/m^2^ Post COVID-19 (mean ± SD): 9.00 ± 3.67 months *IG*: n: 111; 16 ♂, 95 ♀ Age (mean ± SD): 46.76 ± 12.03 y BMI (mean ± SD): 27.64 ± 6.80 kg/m^2^ Post COVID-19 (mean ± SD): 9.04 ± 4.29 months
Nambi et al. [44], 2022, Egypt	Controlled randomized clinical trial	n: 76 Mild COVID-19 *LI:* n: 38 ♂; 3 withdrawals Age (mean ± SD): 63.2 ± 3.1 y BMI (mean ± SD): 23.1 ± 1.6 kg/m^2^ *HI:* 38 ♂; 4 withdrawals Age (mean ± SD): 64.1 ± 3.2 y BMI (mean ± SD): 22.8 ± 1.1 kg/m^2^ Post COVID-19 Sarcopenia
Pancera et al. [45], 2020, Italy	Case report	n: 1 ♂ Severe COVID-19 with ARDS (hospitalization in ICU and invasive mechanical ventilation) Age: 51 y BMI: 17.5 kg/m^2^ Active COVID-19, 10 days post hospital admission
Piquet, et al. [46], 2021, France	Cohort study	n: 100; 66 ♂, 34 ♀ Moderate or severe COVID-19 Age (median ± interquartile range): 66 ± 22 y BMI (mean ± SD): 26.0 ± 5.4 kg/m^2^ Post COVID-19 (mean ± SD): 20.4 ± 10.0 days
Santos et al. [47], 2021, Peru	Case report	n: 1 ♀ Moderate COVID-19 (no hospitalization, weight loss 8 kg, limitation of ADL) Age: 60 y Post COVID-19 28 days
Udina et al. [48], 2021, Spain	Cohort study	n: 33 Moderate or severe COVID-19 *ICU:* n: 20; 10 ♂,10♀ Age (mean ± SD): 58.2 ± 7.9 y *No ICU:* n: 13; 9 ♂, 4 ♀ Age (mean ± SD): 78.4 ± 8.1 y Post COVID

Abbreviations: n: sample size; COVID-19: coronavirus disease; CG: control group; IG: intervention group; ♂: men; ♀: women; SD: standard deviation; y: years; BMI: body mass index; kg: kilograms; m: meters; ICU: intensive care unit; wk: week; LI: low intensity; HI: high intensity; ARDS: acute respiratory distress syndrome; ADL: activities of daily living.

**Table 3:** Intervention, outcomes and results of studies included in the review of the effect of physical activity on strength, respiratory function, fatigue and quality of life in post-COVID-19 and LC adults.

First Author, Year of Publication and Country	Intervention	Outcomes	Results
Liu et al. [40], 2020, China	2 sess/wk; 6 wk; 10 min/sess RMT (Threshold PEP): 3 set * 10 breaths (60% MEP) Cough: 3 sets, 10 active coughs Diaphragm training: 30 breaths, ballast 1–3 kg Stretching Home RMT training: 30 reps/day breaths and coughs	FEV_1_, FVC, FEV_1_/FVC, DLCO 6MWT SF-36 ADL: FIM Scale Anxiety and depression: SAS scale, SDS scale	*Changes from baseline (IG)* ↑* FEV_1_, FVC, FEV_1_/FVC, DLCO ↑* 6MWT ↑* SF-36 ↔ FIM ↓* SAS ↓ SDS *IG* vs. *CG* ↑* FEV_1_, FVC, FEV_1_/FVC, DLCO ↑* 6MWT ↑* SF-36 ↔ FIM ↓* SAS ↓ SDS
Longobardi et al. [41], 2022, Brasil	3 sess/wk; 10 wk Aerobic training: 20–45 min walk, Borg Scale [9,10,11,12,13,14,15,16] Strength training: 6 exercises, 3–4 sets, 10–15 reps, Borg Scale [9,10,11,12,13,14,15,16] Stretching	Handgrip test, 30-STS Modified Balke treadmill exercise protocol, Time-up and go FSS	*Change from baseline* ↑Handgrip test ↑ 30-STS ↑Balke protocol ↑ Time-up and go ↓ FSS
Mayer et al. [42] 2021, USA	2 sess/wk; 8 wk; 40–80 min/sess Aerobic training: 15–45 min, RPE [4,5,6] Strength training: 10–20 min, 10–15 rep, RPE [5,6] RMT: diaphragmatic reeducation	MRC-sum score, handgrip test, lower-extremity unilateral leg press MRC dyspnea scale Time-up and go, 6MWT Eq-5D-5L	*Change from baseline* ↑ MRC-sum score, hand-grip test, leg press ↓ MRC dyspnea scale ↑ Time-up and go ↑ 6MWT ↑ Eq-5D-5L
McNarry et al. [43], 2022, United Kingdom	3 sess/wk; 8 wk; 20 min/sess RMT: 6 sets * 6 breaths (80% SMIP)	MIP, SMIP FITr, TDI Chester step Test Fitness K-BILD Daily activity: wrist accelerometer Mental health: TSRQ	*Change from baseline (IG)* ↑* MIP, SMIP ↑*FITr, TDI ↑* Chester step ↑*K-BILD ↑ Daily activity ↔ TSRQ *IG* vs. *CG* ↑* MIP, SMIP ↑FITr, ↑* TDI ↑ Chester step ↑* K-BILD ↑ Daily activity ↔ TSRQ
Nambi et al. [44], 2022, Egypt	4 sess/wk; 8 wk Aerobic training: 30 min LI (40–60% HR max); HI (60–80% HR max) Strength training: 3 sets/group, 10 reps, 10 RM Stretching and diaphragmatic breathing: 15 min at the beginning and at the end of the training session	Handgrip test Muscle mass: Magnetic resonance SarQoL Kinesiophobia: Tampa Scale	*Change from baseline (LI)* ↑ Handgrip test ↑ Muscle mass ↑SarQoL ↓Kinesiophobia *Change from baseline (HI)* ↑ Handgrip test ↑ Muscle mass ↑SarQoL ↓Kinesiophobia *LI* vs. *HI* ↑* Handgrip test ↔ Muscle mass ↑* SarQoL ↓* Kinesiophobia
Pancera et al. [45], 2020, Italy	1 sess/day; 25 days; 20–45 min/ sess Aerobic training: cycloergometer lower/upper extremity; 20–30 min Strength training: 3 set, 8–10 reps, 50–70% 1 RM RMT (Threshold PEP): 20 min, 10 cm H_2_O Neuromuscular electrical stimulation: 30 min quadriceps, 15–20 mA	MIP, MEP MRC-sum score BMI, quadriceps circumference SPPB BID EuroQoL ADL: BI	*Change form baseline* ↑ MIP, MEP ↑ MRC-sum score ↑ BMI, quadriceps circumference ↑ SPPB ↓ BID ↓EuroQoL ↑ BI
Piquet et al. [46], 2021, France	2 sess/day; 5 days/wk; 20 min/sess Submaximal aerobic training: cycloergometer Strength training: strengthening with body weight exercises (sit-to-stand, tiptoe stands, squats), elastics, and weights, 3 sets, 10 reps for each exercise RMT: controlled diaphragmatic breathing, with work on the inspiratory and expiratory times	Handgrip test: left/right hand Strength upper extremity ADL: BI	*Change from baseline* ↑* Handgrip test left hand ↑*Handgrip test right hand ↑* Strength upper extremity ↑* BI
Santos et al. [47], 2021, Peru	3 sess/wk; 5 wk; 11–75 min/sess Aerobic training: Coordination/balance exercises Strength training: resisted strength TENS CDTM Kinesitherapy: Passive/Active Stretching Manual therapy: Maitland Concept	Daniels strength test Balance: Unipodal Station Pain test: numeric pain scale Mobility: ROM	*Change from baseline* ↑ Daniel’s test ↑Unipodal Station test ↓ Numeric pain scale ↑ ROM
Udina et al. [48], 2021, Spain	7 sess/wk; 10 days, 30 min/sess Aerobic training: cycloergometer, stairs and walking, 5–15 min, Modified Borg Scale [3,4,5] Strength training: 2–4 exercises, 2 sets, 10 reps, 30–80% 1 RM Balance: 2 exercises (static and dynamic)	Strength lower extremity SPPB 6MWT Walking speed FAC Balance ADL: BI	*Change from baseline (ICU)* ↑* Strength lower extremity ↑* SPPB ↑* 6MWT ↑* Walking speed ↑* FAC ↑* Balance ↑* BI *Change from baseline (no-ICU)* ↑* Strength lower extremity ↑* SPPB ↑* 6MWT ↑* Walking speed ↑* FAC ↑* Balance ↑* BI *ICU* vs. *No-ICU* ↑ Strength lower extremity ↑* SPPB ↑ 6MWT ↑ Walking speed ↔ FAC ↑ Balance ↔ BI

Abbreviations: ↑: increase; ↓: decrease; ↔: without change; *: statistically significant changes (*p* < 0.05); sess: session; wk: week; min: minutes; RMT: respiratory muscle training; PEP: positive expiratory pressure; MEP: maximal expiratory pressure; Kg: kilograms; reps: repetitions; FEV_1_: forced expiratory volume in the first second; FVC: forced vital capacity; DLCO: diffusing lung capacity for carbon monoxide; 6MWT: 6-min walk test; ADL: activities of daily living; FIM: functional independence measure; SAS: self-rating anxiety scale; SDS: self-rating depression scale; IG: intervention group; CG: control group; 30-STS: 30 s sit-to-stand test; FSS: Fatigue Severity Scale; RPE: rate of perceived exertion; MRC: Medical Research Council; Eq-5D-5L: EuroQoL 5 dimensions 5 level; SMIP: sustained maximal inspiratory pressure; MIP: maximal inspiratory pressure; FITr: fatigue index time respiratory; TDI: transition dyspnea index; K-BILD: interstitial lung disease quality of life; TSRQ: Treatment Self-Regulation Questionnaire; LI: low intensity; HI: high intensity; HR: heart rate; RM: maximum repetition; SarQoL: sarcopenia quality of life; cm H_2_O: centimeters of water; mA: milliamps; BMI: body mass index; SPPB: Short Physical Performance Battery; BID: Barthel based on dyspnea; BI: Barthel index; TENS: transcutaneous electrical nerve stimulation; CDTM: Cyriax deep transverse massage; ROM: range of movement; FAC: Functional Ambulatory Category; ICU: intensive care unit.

## Data Availability

Not applicable.
